# Editorial: Year in review: discussions in general cardiovascular medicine

**DOI:** 10.3389/fcvm.2023.1341650

**Published:** 2023-12-05

**Authors:** Riccardo Accioli, Viola Salvini, Junjie Xiao, Pietro Enea Lazzerini, Leonardo Roever, Maurizio Acampa

**Affiliations:** ^1^ Department of Medical Sciences, Surgery and Neurosciences, University of Siena, Siena, Italy; ^2^ Institute of Cardiovascular Sciences, Shanghai Engineering Research Center of Organ Repair, School of Life Science, Shanghai University, Shanghai, China; ^3^ Department of Clinical Research, Brazilian Evidence-Based Health Network, Uberlândia, Brazil; ^4^ Gilbert and Rose -Marie Chagoury School of Medicine, Lebanese American University, Beirut, Lebanon

**Keywords:** coronary artery disease, cardiac hypertrophy, abdominal aortic disease, inflammation, cardiovascular medicine

**Editorial on the Research Topic** Year in review: discussions in general cardiovascular medicine

This Research Topic sought to ignite meaningful discussions and enhance our understanding of the advancements and perspectives presented in selected articles from the field of Cardiovascular Medicine in 2022. These articles garnered notable attention throughout the year especially addressing various topics, including coronary artery disease (CAD), cardiac hypertrophy and abdominal aortic disease (AAA).

The mortality rate associated with CAD has declined over recent decades; however, it remains a leading cause of death worldwide, imposing a significant economic burden (1). One of the most important aspects in this setting is represented by refractory angina pectoris, a clinical condition characterized by persistent symptoms lasting beyond 3 months, even with the escalation of pharmacotherapy, including second- and third-line pharmacological agents, as well as bypass grafting, stenting, or percutaneous coronary intervention (PCI) of chronic total coronary occlusion (2, 3). Since the number of subjects with CAD who develop refractory angina pectoris despite optimal medical treatment including exercised based cardiac rehabilitation is growing (4–6), it is of great scientific interest to discover new therapeutic strategies in this field. To better comprehend these aspects, Chen et al. conducted a 20-year (2003–2022) bibliometric analysis, highlighting the potential role of novel treatment methods, including spinal cord stimulation, enhanced external counterpulsation, stem cell therapy, and the coronary sinus reducer. However, despite these advancements, the absence of high-quality randomized controlled trials, long-term evidence of benefits, and cost-effectiveness studies limits their use in refractory angina pectoris. For these reasons, the authors emphasize the need for further research to better understand their potential impact on refractory angina outcome, and, more generally, on CAD outcome.

In this regard, it is worth highlighting how a significative portion of cases of CAD, up to 15%, is due to nonobstructive coronary artery myocardial infarction (MINOCA), defined as myocardial infarction in presence of coronary artery stenosis <50% at angiography (7, 8). The absence of significant coronary stenosis highlights the role of non-atherosclerotic thrombogenic mechanisms, in which the use of antiplatelet therapy may not be efficacious, given the unclear relationship between certain etiologies and platelet function that could negatively impact the final outcome (9). On this important topic, Chen et al. provide an important viewpoint regarding principal aspects of this phenomenon, including a discussion of the types of MINOCA antiplatelet drugs, possible etiologies, and interesting clinical perspectives about MINOCA guidelines and clinical studies. In particular, the authors show the lack of convincing evidence to demonstrate the beneficial effect of dual antiplatelet therapy in the presence of small plaque rupture in non-significant stenosis and non-thrombotic coverage. The absence of a clear indication for antiplatelets on one side suggests the importance of conducting appropriate study populations and prospective randomized controlled trials to better evaluate the impact of antiplatelets agents; on the other hand, it strengthens the use of statins and angiotensin-converting enzyme inhibitor/angiotensin II receptor antagonist, now the only drugs able to reduce mortality in MINOCA patients. Lastly, the authors emphasize the importance of comprehending the underlying cause. This last point is crucial in antiplatelet responsiveness, especially when platelets are not involved in the phenomenon genesis (10). An important contribution to this non-thrombotic state is given to the discrepancy between oxygen demand/offer, for example during cardiac hypertrophy. In this scenario, the role of systemic inflammation in cardiovascular diseases is increasingly well-known. Cardiac hypertrophy, characterized by an increase in cardiomyocyte size (11), is a physiological adaptive response to various stimuli, such as exercise and pregnancy (12). Nevertheless, it can also be caused by pathological factors, such as increased interstitial fibrosis, cell death, cardiac dysfunction and the release of proinflammatory cytokines (13, 14). An important view about this phenomenon is reported by Zhang et al. In their review, the authors describe the role of toll-like receptors (TLRs), innate immune response receptors, as a key factor in cardiovascular diseases. TLRs are a family of pattern recognition receptors that can identify pathogen-associated molecular patterns (PAMPs) and damage-associated molecular patterns (DAMPs). TLRs interact with their ligands and co-receptors to induce the expression of numerous inflammatory factors and inflammatory cell infiltration into the heart, leading to cardiac hypertrophy and heart failure through various inflammatory pathways (Zhang et al.). The TLRs more closely related to cardiac hypertrophy are TLR2, TLR3, TLR4, TLR5, TLR7/8 and TLR9. Currently, there is little evidence in the literature on the role of TLR7/8 and TLR9 in cardiac hypertrophy, in particular on how they mediate inflammatory pathways and heart disease. Importantly, excessive activation of TLRs can lead to chronic inflammation and autoimmune diseases (15, 16). Future perspectives could develop drugs and methods to balance TLRs signaling, considering molecular target therapy against TLRs. Some of the TLR-based agonist and antagonist agents have been shown to be effective in preclinical models and are now entering clinical trials (17, 18). Overall, the goal is to understand the mechanism of the TLRs-mediated inflammatory response in cardiac hypertrophy and to identify potential therapeutic targets through TLRs' downstream and upstream signaling pathways.

Lastly, inflammation could involve not only the heart but also the vessels. In this regard, an important condition that influences the outcome of general population is represented by aortic disease. Abdominal aortic aneurysm (AAA) is an inflammatory vascular disease with associated high disability and mortality. Risk factors for the development of this disease are old age, high blood pressure, male sex, aortic atherosclerosis and smoking (19–22). Thanks to the improvement of screening techniques, the incidence of AAA increases annually. The progression and pathophysiology of AAA are characterized by inflammatory destruction. As seen in the review by Ling et al. the gut microbiota, an “invisible organ”, can contribute to the formation and progression of AAA by directly or indirectly promoting the inflammation of the vascular wall via release of intestinal metabolites, in turn activating TLRs and cell-mediated immunity (23), responsible for intensified arterial wall remodelling (24). The imbalance between pathological and symbiotic bacteria in the gut can lead to changes in immune development and inappropriate inflammatory responses, but it is not yet known whether this imbalance is the cause or result of AAA (Ling et al.). Gut probiotics, antibiotics, immune modulators, nitric oxide, cholesterol-lowering drugs, gut microbiota transplantation, through the modulation of the vascular inflammatory response, could represent new therapeutic perspectives for AAA (25–29).

In conclusion, this Research Topic has not only facilitated insightful discussions but has also enriched our comprehension of pivotal developments and perspectives in Cardiovascular Medicine during 2022. While highlighting emerging therapeutic strategies, these articles emphasize the imperative for further research, to provide continued advancements in understanding, diagnosis, and treatment strategies, ultimately contributing to improved patient outcomes.
